# Role of Food Texture, Oral Processing Responses, Bolus Properties, and Digestive Conditions on the Nutrient Bioaccessibility of Al Dente and Soft-Cooked Red Lentil Pasta

**DOI:** 10.3390/foods13152341

**Published:** 2024-07-25

**Authors:** Milagros Arnal, Lucía Salcedo, Pau Talens, Susana Ribes

**Affiliations:** Instituto Universitario de Ingeniería de Alimentos—Food UPV, Universitat Politècnica de València, Camino de Vera s/n, 46022 Valencia, Spain; miarsa@upv.es (M.A.); lsalmar@upv.es (L.S.); surillo@upv.es (S.R.)

**Keywords:** pulses, food oral processing, bolus characteristics, in vitro digestion, starch digestibility, protein digestibility

## Abstract

The purpose of this study was to assess the impact of food texture, oral processing, bolus characteristics, and in vitro digestive conditions on the starch and protein digestibility of al dente and soft-cooked commercial red lentil pasta. For that, samples were cooked as suggested by the provider and their texture properties were promptly analysed. Then, normal and deficient masticated pasta boluses were produced by four healthy subjects, characterised in terms of their oral processing, bolus granulometry, texture and viscoelastic properties, and finally subjected to static in vitro digestion, according to the INFOGEST consensus for both adults and the older adult population. Normal masticated boluses exhibited greater saliva impregnation and lower proportions of large particles, hardness, and stiffness than deficient masticated boluses. Likewise, insufficiently masticated al dente–cooked pasta boluses caused a delay in oral starch digestion owing to the larger particles attained during food oral processing, while reduced intestinal conditions in the elderly only interfere with the release of total soluble proteins in all samples. This work evidences the importance of considering the initial texture of products, oral capabilities, processing behaviour, and physical and mechanical properties of food boluses in digestion studies, opening new prospects in designing pulse-based foods that meet the nutritional requirements of the world’s population.

## 1. Introduction

In the 20th century, the world’s population was three times smaller than it is today and could reach 9.7 billion people by 2050. However, a demographic shift in the age structure of the world’s population has been noticed in recent decades owing to the rising life expectancy and declining mortality and fertility. In this context, the population over 65 years old is projected to increase from 10% in 2022 to 16% in 2050 [1].

Nutrition is considered a key factor in promoting healthy ageing since many physiological changes, including chewing declines, muscle mass loss, and gastrointestinal disorders, take place during ageing [2,3]. In this regard, pulses are good sources of digestible starch, protein, fibre, minerals, and bioactive compounds [4], making them valuable products for the elderly. They can be employed in dietary strategies to promote positive health benefits and diminish the prevalence and risk of developing chronic diseases in the population [5]. Nevertheless, their consumption remains low due to the excessive intestinal problems provoked, sensory issues, and long cooking times required [4,6]. 

The incorporation of pulse-based ingredients in staple foods can be an effective strategy to raise pulse consumption without modifying the eating habits of consumers. In this context, a growing use of legume flour for pasta preparation has been observed in recent years [4]. Pulse-based pasta is an easy-to-prepare staple with an improved nutritional and sensory profile compared to conventional pasta [7]; but these nutritional benefits could be particularly limited in the elderly due to changes in the masticatory performance, as well as certain gastrointestinal parameters [8,9]. 

As a result, the design of texture-modified products to meet the nutritional needs and oral capabilities of specific populations has increased over the last few years [10,11,12,13]. This challenge requires a deep understanding of the oral processing behaviour and whole digestive process of foods, with special attention to food bolus properties. In this sense, it is well-known that oral impairments lead to the preparation of insufficiently fragmented boluses that may impact digestion, increasing the risk of malnutrition [14,15]. Furthermore, the viscoelastic and rheological properties of food boluses are associated with foods’ texture, fragmentation, and safe-swallowing [10,16,17], influencing their subsequent digestive steps and bioaccessibility of nutrients. Previous studies have evaluated the granulometric, textural, and rheological features of ready-to-swallow boluses, as well as the effect of gastrointestinal declines on the nutrient digestibility of several food commodities [3,15,18]. Nevertheless, no studies have simultaneously reported the consequences of food textures, oral processing, bolus mechanical features, and diverse digestive scenarios on the nutrient bioaccessibility of pulse-based pasta.

Thus, this work aimed to determine the effect of food texture, oral processing responses, bolus properties, and digestive conditions on the starch and protein digestibility of commercial red lentil pasta. To this end, the texture of al dente and soft-cooked pasta samples, their oral processing behaviour, as well as the particle size distribution, texture, and viscoelastic features of normal and deficient chewed pasta boluses were evaluated. Additionally, the starch and protein bioaccessibility of pasta samples after simulating the physiological digestive conditions of adults and seniors were determined.

## 2. Materials and Methods

### 2.1. Materials and Reagents

Commercial fusilli pasta used in this study was made from 100% red lentil flour (Andriani Spa, Bari, Italy). The nutritional composition of the pasta was 1.7% fat, 50.0% carbohydrates, 7.6% dietary fibre, 26.0% proteins, and <0.01% salt.

Digestion enzymes, including salivary α-amylase (A1031) from human saliva, pepsin (P7012) from gastric porcine mucosa, pancreatin (P7545) from porcine pancreas, and porcine bile extract (B8631), were supplied by Sigma-Aldrich Co. (St. Louis, MO, USA). Bovine serum albumin (BSA), L-Tyrosine, sodium dodecyl sulphate (SDS), L-Leucine, trinitrobenzenesulfonic acid (TNBS), D-glucose, and 3,5-dinitrosalicylic acid (DNS) used in analytical assays were also provided by Sigma-Aldrich Co. (St. Louis, MO, USA). All other reagents were acquired from Scharlab, S. L. (Barcelona, Spain).

### 2.2. Pasta Cooking 

Fusilli pasta was cooked in boiling distilled water (ratio 1:20, *w*/*v*) following the specifications of the provider to achieve al dente–cooked (DC) and soft-cooked (SC) pasta. After cooking, samples were drained and kept for 5 min at room temperature until further analysis. Two different batches of DC and SC pasta were prepared. 

### 2.3. Texture Analysis of Cooked Pasta 

The texture properties of DC and SC pasta were measured using a TA-TX2 texture analyser (Stable Micro Systems, Surrey, UK). Samples were placed on the texture analyser platform and cut crosswise with a light knife blade probe (A/LKB) at a test speed of 1 mm/s. For each type of cooked pasta, twenty repeated measurements were done. Cutting work, defined as the area under the curve of force vs. time, and cutting force, described as the maximum force, were calculated with Exponent software Version 6.2 (Stable Micro Systems Ltd., Godalming, UK) [19,20].

### 2.4. Preparation of Pasta Boluses and Oral Processing Patterns

The boluses employed in this study were produced by four volunteers (three females and one male) with ages ranging from 20 to 50 years, good oral health, and natural dentition. For preparing in vivo normal masticated (NM) boluses, volunteers were encouraged to chew 6.0 ± 0.1 g of pasta as usual and to spit out the bolus when feeling the need to swallow. After that, the mean number of chews recorded while obtaining in vivo NM boluses was decreased to 50% to obtain in vivo deficient masticated (DM) boluses [3,15]. These boluses are frequently found in seniors with poor oral healthcare and are marked by a high proportion of large particles and a low degree of food decomposition [14]. 

Eight boluses of DC and SC pasta per mastication condition were prepared to evaluate the oral processing responses. To this end, the number of chews and the chewing time after the chewing sequence were registered. To estimate the amount of saliva uptake from all pasta boluses, the weight of the sample offered to each participant was subtracted from the expectorated bolus [15,21]. It is noteworthy that all in vivo boluses were formed, expectorated, and recovered in sterile containers prior to conducting each test.

### 2.5. Pasta Boluses Characterisation

#### 2.5.1. Granulometric Tests of Pasta Boluses

The granulometric evaluation of in vivo pasta boluses was done by manual dry sieving as described by Blanquet-Diot et al. [8], with minor modifications. To run these assays, 8 boluses of DC and SC pasta per mastication condition were employed. They were put on a nylon mesh of 0.1 mm, cleaned with water, drained for 30 min at 37 °C, and sieved with a soft brush on a pile of 11 sieves with apertures of 10.0, 8.0, 5.0, 4.0, 3.2, 1.0, 0.71, 0.5, 0.355, 0.25, and 0.125 mm (Mecánica Científica, S.A., Madrid, Spain). Finally, results were expressed as indicated by Blanquet-Diot et al. [8] and Peyron et al. [14]. 

#### 2.5.2. Texture Analysis of Pasta Boluses

Bolus texture was characterised following the methodology described by Ribes et al. [15]. Measurements were performed on a TA-TX2 texture analyser (Stable Micro Systems, Surrey, UK). A textural profile analysis (TPA), utilising a double compression cycle test, was performed with a 20 mm cylindrical probe in 24 DC and SC pasta boluses per mastication condition. Each bolus was compressed to 70% at a rate of 3 mm/s and, finally, TPA parameters (hardness, adhesiveness, and cohesiveness) were calculated by using Exponent software (Stable Micro Systems Ltd.).

#### 2.5.3. Viscoelastic Analysis of Pasta Boluses

Viscoelastic characteristics of in vivo pasta boluses were measured in a Kinexus Pro + Rheometer (Malvern Instruments Ltd., Westborough, MA, USA) with rSpace for Kinexus software version 2.0.2 and a Peltier temperature controller. A PLC61/PU40 parallel plate design, with a 4-mm gap, was utilised to conduct non-linear and linear viscoelastic tests at 37 °C. 

Eight boluses of DC and SC pasta per mastication condition were prepared to run the tests. Then, each bolus was immediately placed on the lower geometry, protected with the accessory provided by the supplier to reduce moisture loss during tests, and kept at rest for 180 s for temperature stability and structure relaxation. The limit of the linear viscoelastic region (LVR) and the non-linear viscoelasticity of each pasta bolus was determined by performing a large amplitude oscillatory shear (LAOS) test according to Ribes et al. [15]. Concisely, strain sweep tests were carried out in a strain range from 0.01% to 100% at 1 Hz. To establish the flow point (G′ = G′′), stress sweep assays were run from 0.01 up to 100 Pa at 1 Hz. Finally, the linear viscoelastic properties of each pasta bolus were evaluated with a small amplitude oscillatory shear (SAOS) test from 0.1 to 10 Hz at 1% strain. The rSpace for Kinexus software was then employed to determine the viscoelastic parameters according to Ribes et al. [15]. The stress at which the G′ value decreased from 100% to 90% of its plateau data was used to determine the limit of the LVR [22]. 

### 2.6. In Vitro Digestion Tests of Pasta

**Figure 1:**
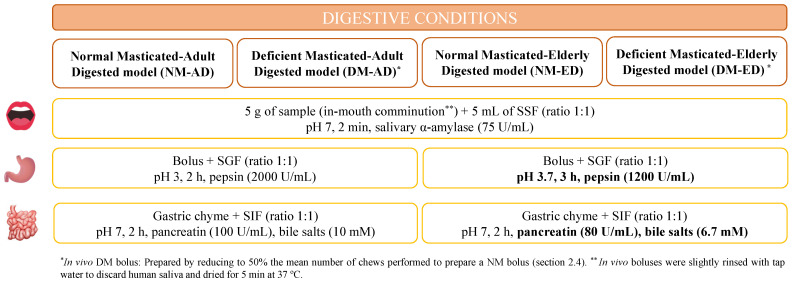
Oro-gastrointestinal conditions of the four models were used to imitate the digestive conditions that could occur in adults and older adults.

Different oro-gastrointestinal conditions were employed to digest pasta samples (Figure 1), having a total of four models. The standardised INFOGEST protocol designed by Brodkorb et al. [23] was used to simulate the gastrointestinal digestion of healthy adults and adults with oral impairments, with minor modifications. In this sense, in vivo NM and DM boluses were employed. They were produced as indicated in Section 2.4, slightly rinsed with tap water to discard human saliva, drained for 5 min at 37 °C, and transferred to falcon tubes until use. These digestion models were defined as the (i) normal masticated–adult digested model (NM-AD) and (ii) deficient masticated–adult digested model (DM-AD). Conversely, the static in vitro INFOGEST digestion model adapted to older populations [24] was employed for evaluating the contribution of the different oro-gastrointestinal changes observed with ageing; but in vivo NM and DM boluses were also used and prepared as above-mentioned. Hence, these two other digestion models were described as the (i) normal masticated–elderly digested model (NM-ED) and (ii) deficient masticated–elderly digested model (DM-ED).

Simulated digestion tests were run in duplicate at 37 °C under continuous mixing (40 rpm) by utilising an incubation cabinet (JP Selecta, S.A., Barcelona, Spain) and a rotary blender (Intell-Mixer™ RM-2, ELMI Ltd., Riga, Latvia). Upon completion of the gastric phase, an aliquot of 1 mL was extracted from every falcon tube previously adjusted to pH 7 with NaOH (1 M) to restrain enzymatic activity. After the intestinal stage, samples were subjected to 98 °C for 5 min to restrain the enzymatic reactions of this phase and immediately chilled with ice. Blank samples (containing simulated fluids, enzymes, and bile salts but no pasta) were prepared and used in each digestion model. Lastly, all samples were centrifuged (8000× *g*, 4 °C, 10 min) and supernatants were maintained at −20 °C for posterior tests. 

#### 2.6.1. Starch Digestion Products of Pasta 

The starch digestibility of pasta products was assessed by determining the reduced sugar content using the DNS method [25]. Data were reported as mg D-glucose/g of the sample (dry basis, d. b.).

#### 2.6.2. Protein Digestibility of Pasta

The proteolysis extent of pasta was established according to Gallego et al. [26] by measuring the total soluble protein content based on Bradford methodology [27], TCA-soluble peptides by using 5% TCA (*w*/*v*) [28], and free amino groups following the TNBS method [29]. Tests were run in triplicate and results were expressed as mg/g of sample (d. b.).

### 2.7. Statistical Tests

Statistical analyses were performed with Statgraphics Centurion XVIII software (Statgraphics Technologies, Inc., The Plains, VA, USA). To analyse the differences between the granulometry of DC-NM and DC-DM boluses and SC-NM and SC-DM boluses, a one-way repeated measures ANOVA test, followed by the Tukey–Kramer post hoc test for the mean comparison at the 5% significance level, was employed. Moreover, a one-way ANOVA test, followed by a Tukey–Kramer post hoc test for a mean comparison at the 5% significance level, was run to evaluate the differences between the texture of DC and SC pasta, as well as among d_50_, viscoelastic, and texture values of all NM and DM pasta boluses, and nutrient digestibility of samples subjected to diverse digestive scenarios. 

## 3. Results and Discussion

### 3.1. Texture Properties of Cooked Pasta 

**Figure 2:**
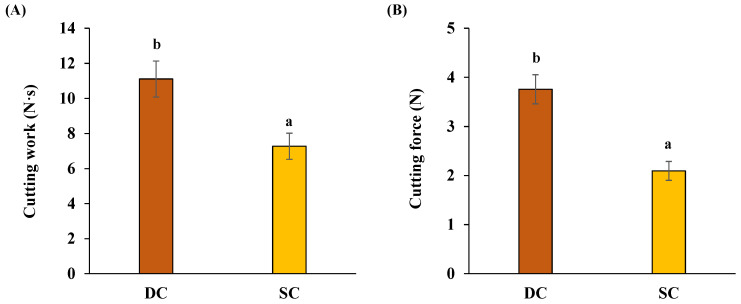
Texture properties of al dente (DC) and soft-cooked (SC) pasta: cutting work (**A**) and cutting force (**B**). Values are expressed as mean (n = 20) ± SD. Different lower-case letters denote significant (*p* < 0.05) differences between different cooked pasta samples.

The texture of cooked pasta is considered one of the most critical parameters when evaluating its quality and acceptance by consumers [19,30]. Figure 2 shows the texture properties of both DC and SC pasta. There were significant differences between samples in terms of cutting work (area under the curve of force vs. time) and cutting force. In this sense, SC pasta showed significantly lower values of cutting work and cutting force than DC pasta due to the longer cooking time used. As the cooking duration progressed, pasta absorbed more water and became less firm [31], also reducing the cutting force. It is important to mention that cutting force values can be correlated with the level of resistance to the first bite [32]. This is crucial when investigating the oral processing of foods given that the chewing time and number of strokes were correlated with different bolus characteristics such as hardness, firmness, and saliva content, among others [33].

### 3.2. Oral Processing Patterns of Pasta

Food oral processing is the first stage of food digestion where products are fragmented into smaller particles due to mastication and saliva impregnation to form cohesive and safe-swallowable boluses [34,35].

**Figure 3:**
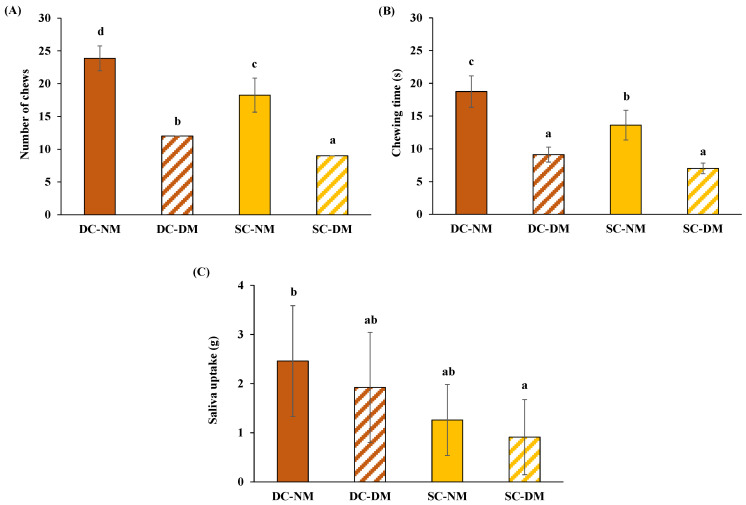
Oral processing patterns of pasta after in vivo normal and deficient chewing: number of chews (**A**), chewing time (s) (**B**), and saliva uptake (g) (**C**). Values are expressed as mean (n = 8) ± SD. Different lower-case letters denote significant (*p* < 0.05) differences among pasta boluses. DC-NM: al dente–cooked–normal masticated; DC-DM: al dente–cooked–deficient masticated; SC-NM: soft-cooked–normal masticated; SC-DM: soft-cooked–deficient masticated.

Figure 3 presents the data on the oral processing behaviour of cooked pasta during chewing. The chewing time and number of chews differed significantly among pasta samples. As can be seen, DC-NM needed a significantly greater number of chews than SC-NM to obtain a safe-swallowable bolus (Figure 3A); meanwhile, in the case of DM samples, the chew number was decreased to 50% to mimic the oral impairments commonly perceived with ageing [3,15]. It is also important to highlight that SC pasta was cooked for a longer period of time than DC pasta; therefore, differing in their moisture content as previously mentioned. In this sense, Motoi et al. [36] pointed out that products with greater moisture content required fewer chewing cycles to achieve safe-swallowable boluses. 

Regarding the duration of the mastication process, DC-NM showed the highest chewing time owing to the greatest number of chews, whereas SC-DM exhibited the shortest chewing time (Figure 3B). Furthermore, SC-NM needed a significantly shorter chewing time than DC-NM to prepare a safe-swallowable bolus. Positive relations between the texture properties of foods, moisture content, and their oral processing responses have been shown by different research studies. In this context, van der Bilt and Abbink [37] pointed out that the number of chewing cycles was smaller in soft foods than in firm foods. Bolhuis and Forde [38] indicated that harder foods, which require higher cutting forces to be disrupted, would need more masticatory cycles and more time to chew to modify their original texture, as well as to break down native structures and fibres. Finally, Chen et al. [39] pointed out that longer chewing times might be related to the lower moisture content and harder texture of foods. 

Concerning the saliva uptake during food oral processing, there were significant differences observed among the samples. NM boluses presented higher amounts of saliva uptake than DM boluses. Likewise, DC-NM bolus showed the greatest saliva uptake, followed by DC-DM, SC-NM, and SC-DM boluses (Figure 3C). Saliva incorporation depends on the mechanical characteristics, water content, and water absorption capacity of food products [40]. For instance, Ilic et al. [41] observed that lower amounts of saliva uptake were needed to form a swallowable bolus in the case of boiled and steamed potatoes, compared to grilled potatoes, owing to their greater moisture content during cooking. Lastly, it is worth mentioning that samples requiring a high level of lubrication will augment the number of chews needed to broaden the surface area of food particles and moisten the bolus entirely before swallowing [38]. 

### 3.3. Granulometric Properties of Pasta Boluses

Fragmentation by in-mouth comminution is one of the main factors reducing the structure of solid foods during oral processing. Changes in the structure degree by fragmentation can be determined by granulometric analysis [42]. Image analysis, laser diffraction techniques, and sieving methods have been used based on foods and particle size ranges [42,43]. In the present work, dry sieving was employed to determine the granulometric properties of pasta boluses obtained in vivo. 

**Figure 4:**
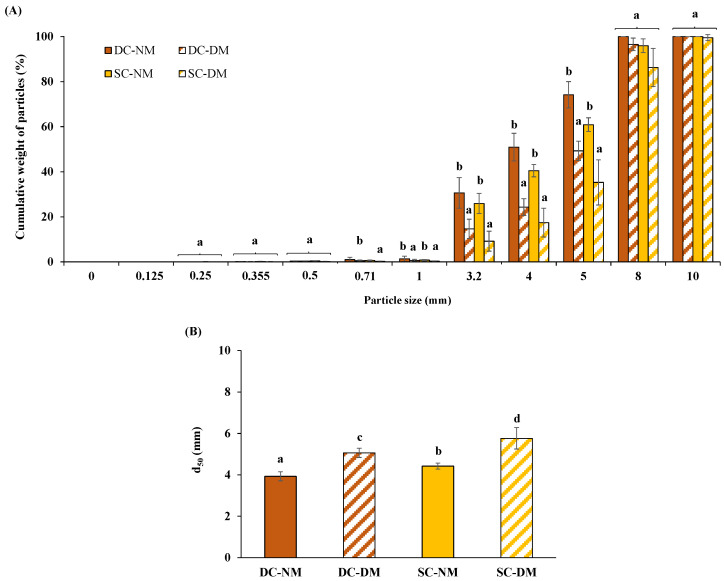
Particle size distribution, indicated as the cumulative weight of particles (%), of all boluses after in vivo normal and deficient chewing (**A**). d_50_ (in mm) of all boluses after in vivo normal and deficient chewing (**B**). Values are displayed as mean (n = 8) ± SD. Different lower-case letters denote significant (*p* < 0.05) differences among pasta boluses. DC-NM: al dente–cooked–normal masticated; DC-DM: al dente–cooked–deficient masticated; SC-NM: soft-cooked–normal masticated; SC-DM: soft-cooked–deficient masticated.

Figure 4 presents the distribution of particle sizes and the d_50_ results of the different normal (NM) and deficient (DM) masticated pasta boluses. As can be observed, NM and DM pasta boluses were significantly different in relation to their particle size distribution, concretely in particles ranging from 5 mm to 0.71 mm. As expected, NM pasta boluses exhibited lower contents of large particles compared to DM pasta boluses (Figure 4A). Furthermore, the d_50_ values of DC-NM and SC-NM pasta boluses were significantly lower compared to both DM boluses (Figure 4B). The latter suggests the formation of poorly disintegrated in vivo pasta boluses when mimicking deficient mastication (Section 2.4). Swallowable boluses produced by seniors often contain a higher proportion of large particles and less food structural fragmentation, especially when complete denture wearers are used [14]. A study on chewing that was conducted with 20 seniors, some with satisfactory dental status and others with poor dental status, showed that food boluses produced by the group with poor dental status had a greater proportion of large particles [10]. Likewise, Mishellany-Dutour et al. [44] pointed out that particle sizes of carrot and groundnut boluses prepared by aged denture wearers were higher than those of aged dentate participants.

Finally, it is also important to highlight that particle fragmentation and bolus formation can vary depending on changes in the initial food texture [45]. Thus, the significantly higher d_50_ values noted in SC pasta boluses compared to DC pasta boluses could be attributed to the lower firmness (cutting force) and higher water content of SC pasta owing to its longer cooking time (Figure 4B). Results are consistent with those observed by Jalabert-Malbos et al. [46] who indicated that softer foods with higher water content produced ready-to-swallow boluses with many large particles. 

### 3.4. Texture Properties of Pasta Boluses

Mastication and insalivation are crucial in the texture features of boluses during oral processing [47]. The texture parameters of normal (NM) and deficient (DM) masticated pasta boluses are presented in Table 1. Hardness was significantly affected by mastication. In this sense, DC-DM was the hardest pasta bolus, which was followed by SC-DM, SC-NM, and DC-NM. Moreover, both NM pasta boluses showed lower hardness values than DM boluses because of the greater number of chewing cycles performed, and saliva incorporated. In populations with oral deficiencies, the initial food pieces are not completely disintegrated, resulting in harder food boluses that may worsen with the absence of saliva [14]. 

Concerning pasta bolus adhesiveness, DC-NM showed significantly higher values compared to DC-DM. This difference could be attributed to the mechanical impact of mastication and saliva incorporation [18,47]. These findings fall in line with those reported by Blanquet-Diot et al. [8] while determining the texture of NM and DM wholemeal chewed pasta. Moreover, DC boluses exhibited significantly higher adhesiveness than SC boluses. It could be explained by mucin and α-amylase activities due to the greater insalivation of DC boluses during mastication caused by the shorter cooking time and lower water absorption [47]. 

The analysis revealed significant differences in cohesiveness among in vivo pasta boluses. DM boluses showed higher cohesiveness than NM boluses, which is consistent with the findings of Peyron et al. [13] in their study on the texture of NM and DM pork frankfurter boluses. Finally, significantly higher cohesiveness values were noticed in both SC pasta boluses compared to DC pasta boluses probably due to the different water content of cooked pasta, as above-mentioned. 

### 3.5. Viscoelastic Properties of Pasta Boluses

Table 2 exhibits the viscoelastic features of normal (NM) and deficient (DM) masticated pasta boluses, which were obtained from the LAOS tests performed at 37 °C. 

G′_LVR_ is associated with the product stiffness, while Strain_LVR_ and Stress_LVR_ values determine products’ extensibility and stability [15,48]. In the present study, the stability of pasta boluses must be understood as less chewed or degraded boluses. As can be seen, G′_LVR_ values did not significantly differ among pasta boluses. However, DM boluses presented a greater stiff structure (higher G′_LVR_ values) compared to NM boluses. This could be due to the lower number of masticatory cycles performed and less saliva mixing in the DM boluses [49]. These results agree with those reported for bolus hardness (see in Section 3.4 for details). Additionally, DC pasta boluses showed the greatest stiff structure while SC pasta boluses exhibited the lowest stiff structure, probably due to the minor water absorbed by DC pasta during the cooking process. 

Regarding the Strain_LVR_ and Stress_LVR_ viscoelastic parameters, in vivo pasta boluses exhibited significant differences. In this sense, DM boluses exhibited higher Strain_LVR_ and Stress_LVR_ values than NM boluses, indicating that less extensible and chewed/degraded boluses were formed. This tendency was also reported by Ribes et al. [15] while studying the viscoelastic features of NM and DM fresh cheese and turkey cold meat boluses. Lastly, the G′ = G′′ values inform about the rupture of the gel-like framework created between food particles and saliva [50]. Although no significant differences were found among in vivo pasta boluses concerning the G′ = G′′ values, both DM boluses exhibited greater values than NM boluses. This suggests that DM boluses flow later than NM boluses possibly due to less saliva being incorporated into DM boluses during chewing (Section 3.2). Enhanced lubrication and flowability could facilitate the transport of the boluses into the oesophagus, reducing the risk of food particle aspiration and contributing to easy and safe swallowing [17].

Table 3 presents the viscoelastic results of normal (NM) and deficient (DM) masticated pasta boluses recorded from the SAOS tests performed at 37 °C. Noteworthy that these viscoelastic parameters were indicated at a frequency of 1 Hz for a better comparison of the results. No significant differences were observed in the G′, G′′, G*, and η* parameters among in vivo pasta samples. 

Based on the results obtained, all pasta boluses can be described as weak viscoelastic systems since they had higher G′ than G′ values. This fact was also described by Tobin et al. [51] when determining the rheological properties of bolus from different particulate foods. Moreover, G* could be used as an index of the bolus stiffness and rigidity, whereas η* is indicative of the resistance of boluses to flow based on the angular frequency [15]. In this sense, the DC-DM pasta bolus showed the highest G* and η* values, meanwhile, the SC-NM pasta bolus reflected the lowest values. DM boluses also showed slightly higher G* and η* values than NM boluses owing to the lower number of masticatory cycles performed, lower amounts of saliva incorporated into these boluses, and higher hardness values given in Section 3.2 and Section 3.4. According to Witt and Stokes [52], differences in bolus rheology could be explained by changes in the mechanical features of food particles and their capability to uptake saliva, which undergoes hydration, swelling, and/or dissolution. Regarding the Tan δ values, they have been extensively used as a rheological criterion to detect easy-swallowable boluses [12,16,17]. For being classified as easy-swallowable boluses, Tan δ values must be comprised between 0.1 and 1. All in vivo pasta boluses had Tan δ values close to 0.2, suggesting their easy-swallowing. Gibouin et al. [53] obtained similar results while investigating the rheological properties of cereal food boluses.

### 3.6. Digestibility of Pasta Samples

Digestion begins in the mouth where foods are fragmented into smaller particles through chewing, and where the incorporation of saliva turns it into a swallowable bolus [13]. In this sense, it is important to highlight that starch hydrolysis starts in the mouth by the activity of salivary α-amylase and keeps on until the prompt gastric digestion [8,54]. Conversely, proteolysis starts in the stomach due to pepsin activity and acidic conditions and is completed in the small intestine by peptidases and proteases [55]. 

**Figure 5:**
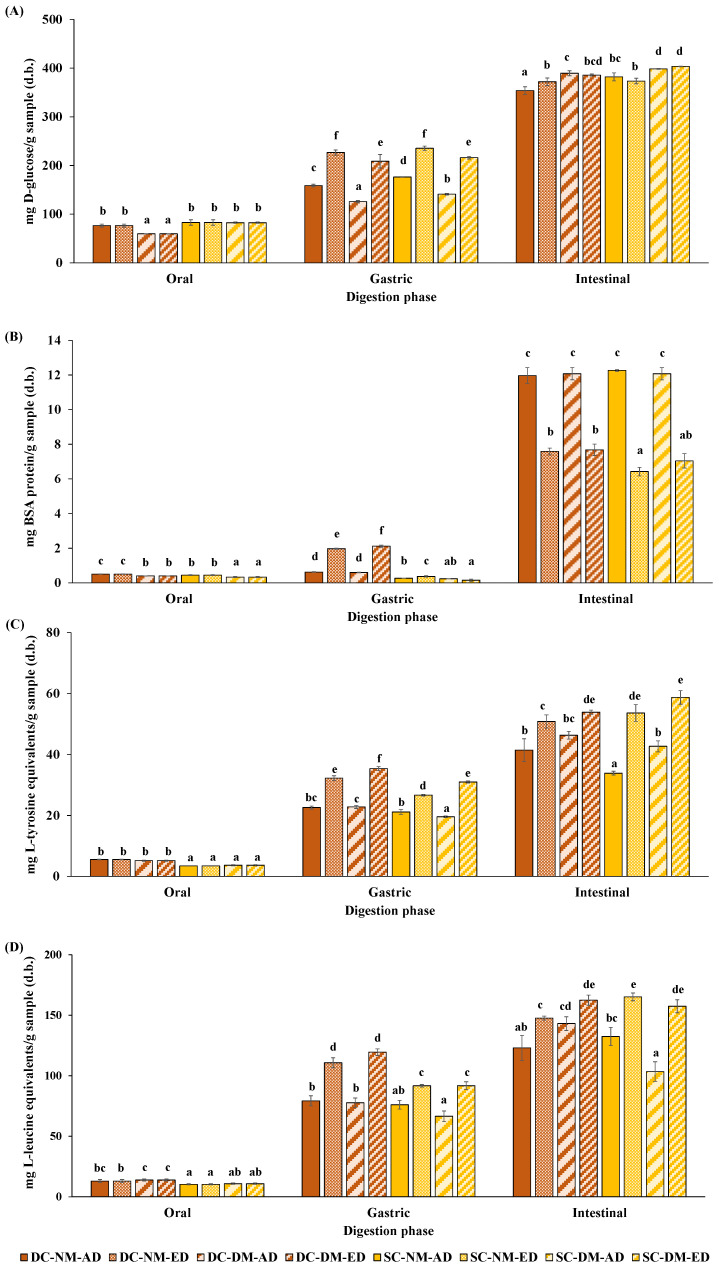
Digestibility of al dente (DC; orange colour) and soft-cooked (SC; yellow colour) pasta: contents of reducing sugars (**A**), total soluble proteins (**B**), TCA-soluble peptides (**C**), and free amino groups (**D**). Mean values (n = 3) ± SD. Lower-case letters showed significant (*p* < 0.05) differences among digested samples within the same digestion phase. NM-AD: normal masticated–adult digested model; DM-AD: deficient masticated–adult digested model; NM-ED: normal masticated–elderly digested model; DM-ED: deficient masticated–elderly digested model.

In the present work, the starch and protein digestibility of DC and SC pasta was estimated by measuring the contents of reducing sugars, total soluble proteins, TCA-soluble peptides, and free amino groups after simulating the oro-gastrointestinal digestion that could occur in adults and seniors (Figure 5). 

Concerning the starch digestibility of samples, a higher content of reducing sugars was reported as digestion advanced. Upon completion of the oral phase, the reducing sugar content of samples significantly differed among digestion models (Figure 5A). In this sense, lower contents of reducing sugars were shown by DC-DM-AD and DC-DM-ED. These results suggest that insufficiently fragmented food boluses prepared by people with oral declines could reduce food-saliva exchanges, delaying oral starch digestion due to the lower surface-to-volume ratio [8,56,57]. In this sense, Blanquet-Diot et al. [8] observed that DM-refined and wholegrain pasta boluses resulted in less saliva impregnation and led to a weak onset of oral digestion. Furthermore, Ribes et al. [56] noted that the level of oral starch digestion increased when having greater proportions of large particles in poorly fragmented bread boluses. Nonetheless, chewing had no significant effect on the reducing sugar content of SC pasta at this stage, which could be attributed to the longer cooking time and consequent softness. During cooking, the starch gelatinises, making it more susceptible to enzymatic breakdown due to swelling, loss of crystalline structure, and thickening of the surrounding matrix [58]. Additionally, low firmness and network destabilisation of pasta provoked by longer cooking times may facilitate the access of enzymes [59]. After the gastric phase, significantly higher amounts of reducing sugars were reported when reproducing the digestive disorders commonly found in seniors (Figure 5A). It could be attributed to the length of this phase (3 h rather than 2 h) and the slightly higher pH employed (pH 3.7 rather than 3.0). Noteworthy that the amylolytic activity of α-amylase is lost below pH 3.5 but its optimal activity is close to 50% at pH 4 [54]. Finally, the highest content of reducing sugars observed after the intestinal phase could be explained by the pancreatic α-amylase activity that supports the amylolytic process [54]. Similar results were reported by Duijsens et al. [4] while studying the in vitro digestibility of lentil-based pasta. However, it is important to mention that no clear trend was observed between digested samples at this stage (Figure 5A).

Regarding protein digestibility, a greater content of total soluble proteins was noted as gastrointestinal digestion progressed (Figure 5B). After the oral phase, significantly higher amounts of total soluble proteins were observed in DC-NM-AD, DC-NM-ED, SC-NM-AD, and SC-NM-ED compared to the models where DM boluses were used (Figure 5B). This could suggest the aggregation or precipitation of proteins due to the interaction with salivary α-amylase since proteolysis is not expected at this stage [26,60]. Upon completion of the gastric phase, significantly higher amounts of total soluble proteins were observed in DC-NM-ED and DC-DM-ED, probably due to the longer length of this phase (3 h rather than 2 h) when simulating the elderly digestive conditions. Conversely, the lower content of total soluble proteins reported by SC-DM-AD and SC-DM-ED could be linked to the presence of greater proportions of large particles reaching the gastric compartment that would hinder the enzymes’ access to the sites of cleavage because of their reduced exposure to the protein surface [61]. Lastly, DC-NM-ED, DC-DM-ED, SC-NM-ED, and SC-DM-ED exhibited significantly lower levels of total soluble proteins at the end of digestion. It could be explained by the reduced pancreatin activity (80 U/mL rather than 100 U/mL) and bile salt concentration (6.7 mM rather than 10 mM) employed when simulating the elderly digestive conditions. Lower pancreatic activities have been linked to deficient digestion processes, which favours protein malabsorption and future nutritional deficiencies [62]. A similar trend was described by Hernández-Olivas et al. [9] while measuring the impact of age-related in vitro gastrointestinal alterations on the protein digestibility of legumes. 

Regarding the TCA-soluble peptides, it is important to indicate that the highest values were recorded at the end of digestion, as the 5% TCA-soluble protein fraction would be constituted by small peptides (<10 amino acid residues), as well as free amino acids [63]. However, no significant differences were found among digestion models upon completion of the oral phase, but slightly lower amounts of TCA-soluble peptides were found in SC samples compared to DC samples (Figure 5C). This decrease could be attributed to protein aggregation through the formation of intra- and intermolecular interactions favoured by longer cooking times [4]. In contrast, the content of TCA-soluble peptides was significantly higher after the gastric and intestinal stages in DC-NM-ED, DC-DM-ED, SC-NM-ED, and SC-DM-ED (Figure 5C). The longer duration of the gastric phase would facilitate the enzyme diffusion, releasing further amounts of TCA-soluble peptides and compensating for the suboptimal intestinal conditions employed [3].

Finally, a higher release of free amino groups was also noted as digestion progressed (Figure 5D). DC-DM-AD, DC-DM-ED, SC-DM-AD, and SC-DM-ED showed greater amounts of free amino groups after the oral stage; but mastication did not clearly influence their release after this stage, probably due to the reduction of the particle size of DM boluses during digestion [64]. Protein aggregation due to intra- and intermolecular interactions resulting from longer cooking times could be responsible for the lower release of free amino groups in SC samples than in DC samples [4]. Moreover, DC-NM-ED, DC-DM-ED, SC-NM-ED, and SC-DM-ED showed significantly greater amounts of free amino groups after the gastric and intestinal phases. This outcome could be explained by the longer duration of the gastric phase that would compensate for the suboptimal conditions used in the intestinal phase, as previously indicated.

## 4. Conclusions

This work demonstrates that the texture of cooked pasta, cooking time, oral processing patterns, and degree of food disintegration reached during chewing play a significant role in the granulometric, texture, and viscoelastic features of red lentil pasta. Moreover, poor chewing has a negative impact on the starch hydrolysis of al dente–cooked pasta upon completion of the oral phase, whereas reduced intestinal conditions in the elderly only interfere with the release of total soluble proteins of al dente and soft-cooked pasta samples. These findings provide useful information about food oral processing responses, physical and mechanical properties of swallowable boluses, and nutrient bioaccessibility in adults and seniors with different oral capabilities, opening new perspectives to design pulse-based foods for specific populations. Nonetheless, further studies emphasising the effect of chewing on bolus features and digestion are needed but should use the in vitro gastrointestinal conditions of the INFOGEST model adapted for older adults.

## Figures and Tables

**Table 1:** Texture features of al dente–cooked and soft-cooked pasta boluses after in vivo normal and deficient chewing. Values are indicated as mean (n = 24) ± SD.

Samples	Hardness (N)	Adhesiveness (N·s)	Cohesiveness (%)
DC-NM	9.72 ± 2.00 ^a^	−0.248 ± 0.169 ^a^	36.38 ± 5.17 ^a^
DC-DM	15.36 ± 2.75 ^b^	−0.082 ± 0.118 ^bc^	41.94 ± 4.21 ^b^
SC-NM	10.57 ± 1.85 ^a^	−0.015 ± 0.095 ^b^	42.74 ± 3.16 ^b^
SC-DM	14.70 ± 2.78 ^b^	−0.001 ± 0.000 ^c^	49.26 ± 2.85 ^c^

Different lower-case letters denote significant (*p* < 0.05) differences among pasta boluses. DC-NM: al dente–cooked–normal masticated; DC-DM: al dente–cooked–deficient masticated; SC-NM: soft-cooked–normal masticated; SC-DM: soft-cooked–deficient masticated.

**Table 2:** Viscoelasticity of al dente–cooked and soft-cooked pasta bolus after in vivo normal and deficient chewing. Values are indicated as mean (n = 8) ± SD.

Samples	G′_LVR_ (Pa)	Strain_LVR_ (%)	Stress_LVR_ (Pa)	G′ = G′′ (Pa)
DC-NM	9591 ± 2654 ^a^	0.28 ± 0.05 ^a^	28 ± 11 ^a^	247 ± 59 ^a^
DC-DM	10,592 ± 2375 ^a^	0.39 ± 0.03 ^b^	42 ± 11 ^b^	265 ± 64 ^a^
SC-NM	8198 ± 1519 ^a^	0.24 ± 0.03 ^a^	20 ± 5 ^a^	217 ± 27 ^a^
SC-DM	8798 ± 1604 ^a^	0.29 ± 0.06 ^a^	26 ± 8 ^a^	255 ± 50 ^a^

Viscoelastic parameters from large amplitude oscillatory shear (LAOS) assay at 37 °C: elastic modulus value at LVR, G′_LVR_; strain value at LVR, Strain_LVR_; stress value at LVR, Stress_LVR_; flow point, G′ = G′′. Different lower-case letters expressed significant (*p* < 0.05) differences among pasta boluses. DC-NM: al dente–cooked–normal masticated; DC-DM: al dente–cooked–deficient masticated; SC-NM: soft-cooked–normal masticated; SC-DM: soft-cooked–deficient masticated.

**Table 3:** Viscoelastic data, obtained from small amplitude oscillatory shear (SAOS) tests performed at 37 °C and recorded at 1 Hz of al dente–cooked and soft-cooked pasta bolus after in vivo normal and deficient chewing. Values are indicated as mean (n = 8) ± SD.

Samples	G′ (Pa)	G′′ (Pa)	G* (Pa)	η* (Pa·s)	Tan δ
DC-NM	8289 ± 2381 ^a^	1980 ± 516 ^a^	8524 ± 2431 ^a^	1357 ± 387 ^a^	0.241 ± 0.018 ^b^
DC-DM	9471 ± 2260 ^a^	2167 ± 417 ^a^	9716 ± 2295 ^a^	1546 ± 365 ^a^	0.231 ± 0.013 ^ab^
SC-NM	7161 ± 1409 ^a^	1629 ± 295 ^a^	7344 ± 1439 ^a^	1169 ± 229 ^a^	0.228 ± 0.009 ^ab^
SC-DM	8558 ± 1322 ^a^	1883 ± 290 ^a^	8763 ± 1347 ^a^	1432 ± 215 ^a^	0.221 ± 0.014 ^a^

Elastic modulus, G′; viscous modulus, G′′; complex modulus, G*; complex viscosity, η*; loss tangent, Tan δ. Different lower-case letters expressed significant (*p* < 0.05) differences among pasta boluses. DC-NM: al dente–cooked–normal masticated; DC-DM: al dente–cooked–deficient masticated; SC-NM: soft-cooked–normal masticated; SC-DM: soft-cooked–deficient masticated.

## Data Availability

The original contributions presented in the study are included in the article, further inquiries can be directed to the corresponding author.
